# The Intrinsic Experience of Tourism Autobiographical Memory on Environmentally Responsible Behavior: A Self-Expansion Perspective

**DOI:** 10.3390/bs15010002

**Published:** 2024-12-24

**Authors:** Junxian Shen, Cora Un In Wong, Hongfeng Zhang, Fanbo Li, Jianhui Chen

**Affiliations:** 1 Faculty of Humanities and Social Sciences, Macao Polytechnic University, Macao 999078, China; p2315267@mpu.edu.mo (J.S.); corawong@mpu.edu.mo (C.U.I.W.); 2 School of Social Sciences, Tsinghua University, Beijing 100190, China; 3 School of Hospitality and Tourism Management, The Hong Kong Polytechnic University, Hong Kong 999077, China

**Keywords:** travel memory, sustainable tourism behavior, psychological richness, self-expansion theory, tourist experience, personality beliefs

## Abstract

The existing literature on environmentally responsible behavior in tourists focuses primarily on the factors that influence this behavior, such as tourists’ attitudes and negative feelings. However, the intrinsic benefits of conservation for individual and societal well-being are often overlooked. Under the theoretical lens of self-expansion theory, this study examined the influence of Chinese tourists’ tourism autobiographical memory on their environmentally responsible behavior using a questionnaire survey (N = 434) with partial least squares structural equation modeling. The result attested that tourists’ self-expansion and psychological richness serially mediate the association between their tourism autobiographical memory and environmentally responsible behavior as a tourist. In addition, the implicit theories of personality moderate the prediction of tourist autobiographical memory on self-expansion. The results provide an additional explanation for environmentally responsible behavior in tourists, with practical implications for marketers and operators in the industry.

## 1. Introduction

The phenomenon of global climate change represents a significant environmental challenge currently confronting the planet (Pörtner et al., 2022). Carbon dioxide emissions are a major cause of climate change, and emissions from tourism are still increasing (Peeters et al., 2024). With the continuing expansion of global tourism, the environmental impact of travel is an issue that requires immediate attention (Han, 2021). Tourist behavior has a major impact on the environmental sustainability of destinations, affecting natural resources, climate change, wildlife, and local communities (Han, 2021; Peeters et al., 2024; Qiu et al., 2023). The promotion of environmentally responsible behaviors in tourists can help mitigate negative impacts such as pollution, habitat destruction, and overconsumption of resources (Confente & Scarpi, 2021). Tourist environmentally responsible behavior (TERB) is any behavior exhibited by tourists in the tourism context that promotes environmental sustainability and the minimization of negative impacts on the environment (Qiu et al., 2023). Fostering such behaviors aligns with the broader goals of sustainable development, ensuring that tourism contributes positively to the environment and society (Su et al., 2020). Previous research on tourist environmentally responsible behavior has primarily focused on factors such as environmental attitudes, awareness, and motivations, as well as demographic characteristics that influence such behaviors (Kil et al., 2014; Lee & Jan, 2015; Luo et al., 2020). While these studies have provided valuable insights, they often overlook some equally crucial individual factors that shape tourists’ actions, particularly the role of personal memories. It has been demonstrated that memory plays a pivotal role in influencing habits, attitudes, motivation, and behaviors (Albarracin & Wyer, 2000; Verplanken & Orbell, 2022); thus, studying the effects of memory also expands on previous research in this field. In addition, previous studies have highlighted a common perception among the general public that environmental protection is often associated with negative results including loss, distress, and inconvenience (Pritchard, 2010; Suárez-Varela et al., 2016). This perception can hinder the adoption of environmentally responsible behaviors, as individuals may be reluctant to engage in actions they believe will reduce their personal happiness (Venhoeven et al., 2013). However, this view overlooks the intrinsic benefits of environmental stewardship, which is closely linked to personal and social well-being. In fact, environmentally responsible behaviors can enhance an individual’s sense of well-being, aligning with broader human motivations to pursue a good life (Binder et al., 2020).

One theoretical framework that offers a promising avenue for addressing these gaps is self-expansion theory (Aron & Aron, 1986). This theory posits that individuals are motivated to enhance their self by incorporating new experiences, knowledge, and relationships into their sense of self (Aron et al., 2004). Autobiographical memory is defined as the capacity to recall personal experiences, encompassing both semantic and episodic information (Conway, 2005; Rubin & Umanath, 2015). Tourism autobiographical memory (TAM) is a construct that assesses the extent to which travel experiences shape a tourist’s life trajectory and serves as a gauge for the enduring influence of travel on an individual’s memory and identity (Jorgenson et al., 2019). With regard to the field of tourism, autobiographical memories of past travels can serve as significant self-expansion opportunities, where individuals assimilate these experiences into their self (Nolan & Schultz, 2015). Such memories can lead to an expanded sense of self that is more inclusive of environmental considerations and possesses a broader perspective on the world (Kim & Chen, 2021; Oishi et al., 2024). As individuals expand their self-boundaries through enriching experiences, they may become more prone to engage in actions that protect the environment, recognizing these actions as part of a broader pursuit of personal and social well-being. This integrated approach provides a more holistic understanding of the motivations behind environmentally responsible behavior, suggesting that it is not merely a matter of sacrifice but also a pathway to a good life (Binder et al., 2020; Wei et al., 2023).

Notwithstanding the growing interest in understanding the psychological drivers of TERB, significant gaps remain in the existing literature. The majority of existing research has concentrated on external factors, including environmental awareness, economic incentives, and regulatory mechanisms (Kil et al., 2014; Lee & Jan, 2015; Luo et al., 2020). While these approaches offer valuable insights, they often overlook the deeper psychological processes that shape an individual’s sustainable tourism behavior. Autobiographical memory has been demonstrated to influence decision-making and behavior in a range of domains (Bluck, 2003); however, it has received limited attention in the context of TERB. This lack of focus is surprising, given that unforgettable travel experiences are often rich, emotionally significant, and enduring, suggesting that they may serve as powerful motivators for TERB. Additionally, there has been little application of self-expansion theory in this context. Understanding how tourism autobiographical memory, through the lens of self-expansion, influences responsible tourism behavior could provide a deeper insight into the psychological drivers of sustainable practices. This approach not only broadens the theoretical framework for studying responsible tourism but also offers practical implications for how past experiences can be utilized to promote more sustainable behaviors among tourists. For example, findings could inform interventions that use memory-based prompts to encourage sustainable behaviors, or experiential marketing strategies that emphasize personal growth and environmental connection. Furthermore, it addresses the broader need to understand how intrinsic motivations, rooted in personal growth and reflection, can contribute to achieving global sustainability goals. To accomplish this, this study conducts an empirical investigation that examines these relationships through data collected from tourists.

## 2. Literature Review and Hypothesis Development

### 2.1. Self-Expansion Theory

The self-expansion theory, originally developed in the context of interpersonal relationships, posits that people are driven to expand their self by incorporating new experiences, knowledge, and social connections into their sense of self (Aron & Aron, 1986). Drawing on this, researchers have further applied it to the area of environmental protection, proposing that self-expansion (SE) can be understood as encompassing three dimensions: the self, humans, and nature (Schultz, 2001). In particular, the self (e.g., an individual’s self-concept, health) is located within the boundaries of humans (e.g., social groups). Similarly, individuals are also embedded within the broad boundaries of nature (e.g., animals and plants) (Nolan & Schultz, 2015). The self-expansion theory posits that each individual possesses distinct personal boundaries pertaining to self, other individuals, and nature, which are shaped by the specific experience they encounter (Schultz, 2001). Research indicates that self-expansion has the potential to counter the limitations of traditional thought and expand established boundaries of self, humans, and nature, thereby facilitating purposeful behaviors such as environmentally responsible behaviors (Tang et al., 2017). The self-expansion theory has been validated in studies of intimate relationships (Harasymchuk et al., 2021), business (Sohail, 2023), and pro-environmental behaviors (Tang et al., 2017). However, it has been less applied to tourism memory. In the realm of tourism, self-expansion can occur as individuals engage in new and diverse travel experiences that broaden their understanding of the world and themselves. These memories, in turn, can influence their attitudes and behaviors, including their approach to environmental responsibility (Cajiao et al., 2023). The present study aims to investigate the association between tourism autobiographical memory and TERB from the perspective of the self-expansion theory.

### 2.2. Tourism Autobiographical Memory and Tourist Environmentally Responsible Behavior

Autobiographical memory is defined as the capacity to recall personal experiences, encompassing both semantic and episodic information (Conway, 2005; Rubin & Umanath, 2015). The function of autobiographical memory is to facilitate the resolution of current problems by enabling the recall of past experiences. This enables individuals to make informed decisions, form connections with others, and maintain a sense of self-continuity (Bluck, 2003). It integrates past experiences into the self-concept, which in turn directs present and future behaviors (Bluck, 2003). Tourism autobiographical memories can create a lasting connection between travelers and the environments they have encountered, influencing their future behaviors (Kim & Chen, 2021). Research has shown that memories of travel can impact tourists’ future travel plans and purchasing decisions (Kim & Chen, 2019; Yin et al., 2017). Moreover, these behavioral influences are enduring. For example, Hu and Wan (2024) have demonstrated that recalling travel memories, even those from long ago, in the context of challenging circumstances, can lead to the generation of new insights.

Studies have shown that personal travel memories influence an individual’s values and actions (Massingham et al., 2019). When tourists reflect on their past travel memories, particularly those involving nature or culturally significant encounters, these memories can influence their attitudes toward environmental stewardship (Buckley, 2022). For example, research indicates that tourists can effectively influence public awareness and the adoption of long-lasting environmentally friendly attitudes and behaviors by evoking “powerful memories” in Antarctica (Cajiao et al., 2023). Tourism autobiographical memories can be characterized by two dimensions: impact and rehearsal. Impact relates to the emotional depth of the memory, while rehearsal pertains to how often the event is recalled, either voluntarily or involuntarily (Jorgenson et al., 2019). Autobiographical memories often evoke strong emotions and a deep connection to the places visited. These memories become part of an individual’s identity, reinforcing a sense of responsibility toward the environments that contributed to their positive experiences (Cajiao et al., 2023). Consequently, individuals who hold vivid, impactful memories of their travels are more likely to engage in TERB (Bahja & Hancer, 2021). Therefore, we posited that:

**H1.**  *Tourism autobiographical memory positively impacts TERB.*

### 2.3. Tourism Autobiographical Memory and Self-Expansion

Self-expansion theory suggests that people seek to enhance their sense of self by incorporating novel experiences, knowledge, and relationships into their self-identity (Aron et al., 2004). When people travel, they often encounter new environments, cultures, and challenges that provide individuals with new insights and understandings of things or people (Oishi et al., 2021). The nature of on-site travel experiences is such that they are ephemeral, providing experiences that are fleeting (Kim, 2010). In contrast, experiences stored in tourism memories are of significant importance, as they can be recalled by travelers during a trip or at other times of need (Jorgenson et al., 2019; Kim et al., 2022). These travel experiences, when encoded as autobiographical memories, become lasting elements of an individual’s identity (Jorgenson et al., 2019). As these memories are recalled, they reinforce the personal growth and expanded perspectives gained during the travels. According to the above discussion, the following hypothesis was put forth:

**H2.**  *Tourism autobiographical memory positively impacts tourist self-expansion.*

### 2.4. Tourism Autobiographical Memory and Psychological Richness

Psychological richness (PR) is characterized by a life filled with varied, complex, and intellectually stimulating experiences and shifts in perspective (Oishi et al., 2020). Oishi et al. (2021) first examined which activities could predict psychological richness, and of the 14 activities examined, travel was the strongest predictor. When tourists reflect on their travel memories, particularly those involving novel and memorable interactions with nature or local communities, these memories can facilitate personal growth and foster new insights into others or nature (Oishi et al., 2020; Oishi & Westgate, 2022). This is due to the fact that such experiences often result in a shift in perspective, leading to a deeper understanding of self and one’s position in the world (Oishi & Westgate, 2022). For example, the study demonstrated that the wellness tourism experience can enhance tourists’ holistic wellness perspectives (Dillette et al., 2021) and facilitate their engagement with the ecological environment, fostering enhanced interaction and improved interpersonal relationships (Liao et al., 2023). This process aligns with the principles of self-expansion theory, which suggests that people can enhance their self by incorporating new experiences into their self (Schultz, 2001). Thus, we hypothesize the following:

**H3.**  *Tourism autobiographical memory positively impacts tourists’ psychological richness.*

### 2.5. Mediating Role of the Self-Expansion and Psychological Richness

Tourist autobiographical memory represents a specific form of long-term memory that can leave a lasting influence on the lives of tourists (Jorgenson et al., 2019). With the passage of time, these memories persist, reinforcing the expansion of the boundaries of the self. This expansion encompasses a heightened awareness and appreciation of the natural environment and the diverse ways in which different peoples and cultures interact with it (Schultz, 2001). In other words, tourist autobiographical memories facilitate tourists’ connections to others and the natural environment, and may contribute to a greater tendency to behave responsibly towards the environment (Buckley, 2022; Schultz, 2001). Individuals who perceive themselves as connected to the well-being of the natural environment they explore and the communities they come into contact with are more inclined to take action to protect and preserve these elements (Fritsche & Masson, 2021; Schultz, 2001). Thus, we hypothesize the following:

**H4.**  *Self-expansion mediates the positive effect of tourism autobiographical memory on TERB.*

Recently, the mediating role of psychological richness has received attention from scholars (Gu et al., 2023). The existing research on travel memories indicates that experiences that are unusual, surprising, and unexpected tend to result in more vivid and enduring memories for individuals than those that are normal, routine, and ordinary (Kim, 2010; Yin et al., 2017), which is consistent with the connotation of psychological richness. Furthermore, the study demonstrated that psychological richness can enhance an individual’s proclivity to engage in pro-environmental behaviors (Wei et al., 2023). This is probably due to the fact that as psychological richness increases, individuals tend to become more open-minded (Oishi et al., 2020), demonstrating greater willingness to endorse social transformations in environmental protection, accept environmentally friendly life concepts and lifestyles, and actively participate in pro-environmental and environmentally responsible behaviors throughout their lives (Poškus, 2018). Thus, we hypothesize the following:

**H5.**  *Psychological richness mediates the positive effect of tourism autobiographical memory on TERB.*

### 2.6. A Serial Multiple Mediation Model

In self-expansion theory, individuals are motivated to expand their self by integrating new experiences, knowledge, and relationships into their self (Aron et al., 2004; Schultz, 2001). This drive is particularly evident in the context of travel, where exposure to new environments, cultures, and perspectives provides rich opportunities for personal expansion (Oishi et al., 2021). As tourists reflect on their past travel experiences, these memories serve as a means of self-expansion, facilitating the integration of diverse perspectives and encounters experienced during their travels, and thus expanding their boundaries of self, human, and nature (Nolan & Schultz, 2015). As these novel and unforgettable memories contribute to self-expansion, they also increase psychological richness (Oishi et al., 2020). This psychological richness deepens the individual’s connection to the environments and cultures encountered. As a result, the boundaries of the self expand to include these new dimensions, creating a more comprehensive and interconnected view of the world, thereby enhancing psychological richness (Oishi et al., 2024). This expanded self-concept, now intertwined with a broader understanding of humanity and nature, facilitates purposeful behaviors, such as environmentally responsible actions (Tang et al., 2017; Wei et al., 2023). The social identity model of pro-environmental behavior also posits that individuals become more willing to take actions that protect and conserve the environments that have become integrated into their self, thereby aligning their actions with their expanded selves and values (Fritsche & Masson, 2021). We thus propose:

**H6.**  *Self-expansion and psychological richness sequentially mediate the positive effect of tourism autobiographical memory on TERB.*

### 2.7. Moderating Role of the Implicit Theories of Personality

Implicit theories of personality (ITP) are defined as the beliefs people hold regarding the nature of personality traits, whether they are fixed and unchangeable or malleable and capable of development over time (Chiu et al., 1997). These theories are typically categorized into two perspectives: entity theory and incremental theory (Chiu et al., 1997). Individuals who subscribe to the entity theory believe that personality traits are inherent and stable, suggesting that people cannot change their basic characteristics significantly. On the other hand, those who subscribe to the incremental theory believe that personality traits can evolve through effort, experience, and personal growth (Yeager et al., 2014).

In the context of tourism, these implicit personality theories may moderate the association between tourists’ autobiographical memory and self-expansion (Mattingly et al., 2019). People who hold an incremental theory believe that their traits can grow and change, and travel experiences are seen as valuable opportunities for personal growth and transformation (Steimer & Mata, 2016). When they reflect on their travel memories, they are more likely to perceive these experiences as contributing to their self-expansion by integrating the various encounters and lessons learned into their evolving sense of self. This belief in the potential for change makes their autobiographical travel memories more powerful in promoting self-expansion. Conversely, those with an entity theory may not perceive their travel experiences as opportunities for significant personal development. They may believe that these experiences do not fundamentally change their personality or sense of self (Yeager et al., 2014). As a result, the potential for these memories to contribute to self-expansion is reduced. Thus, the following hypothesis was proposed:

**H7.**  *The implicit theories of personality moderate the prediction of tourism autobiographical memory on self-expansion.*

**Figure 1:**
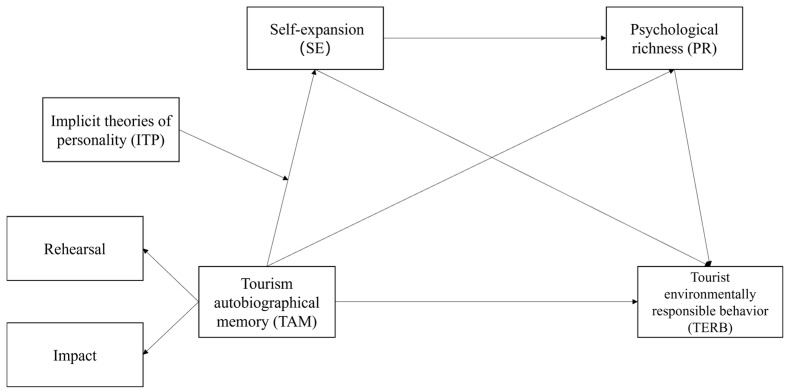
Conceptual model.

The conceptual model is presented in Figure 1.

## 3. Materials and Methods

### 3.1. Measurements

The Tourism Autobiographical Memory Scale was employed to evaluate tourism memory, which was conceptualized as a two-order structure consisting of rehearsal (five items) and impact (three items) (Jorgenson et al., 2019). In accordance with the original scale design, participants were instructed to respond to the scale with the first tourist memory that came to mind (Jorgenson et al., 2019). This scale has been validated in the Chinese context (Zhao & Li, 2022). A lower total score indicates a lower level of rehearsal and impact of the tourist memory.

To measure self-expansion, the Individual Self-Expansion Questionnaire was utilized (Mattingly et al., 2013). The questionnaire consists of five items and has been validated in the Chinese context (Wei et al., 2023). The higher the score, the more the individual’s sense of self had expanded.

In terms of psychological richness, the Chinese version of the Psychologically Rich Life Questionnaire was employed (Oishi et al., 2019), which has been validated by scholars (Wang et al., 2022). The questionnaire utilized 12 items to measure individuals’ psychological richness, with higher scores indicating a higher level of psychological richness.

The tourist environmentally responsible behavior was measured using the full set of four items used by Qiu et al. (2023), with higher scores indicating a higher level of TERB. Implicit personality theories were assessed using three items (Chiu et al., 1997) that were reverse scored, with higher scores indicating that the individual favored the incremental theory and vice versa for the entity theory.

All constructs were rated on a 7-point Likert scale, except for item 5 of the tourism autobiographical memory rehearsal, which followed a 4-point scale as per the original design (Jorgenson et al., 2019). Detailed items and constructs are provided in Appendix A.

### 3.2. Data Collection

In the period between February and April 2024, traveling tourists staying in four hotels in different regions of China (eastern, southwestern, southern, and northern) were invited to participate in an online questionnaire survey through a collaboration with a travel agency to ensure geographic diversity, which aimed to capture a wide range of tourist perspectives and reduce the risk of regional bias. To minimize the environmental impact of the data collection process, and in line with the principles of environmentally responsible behavior, an online survey was used instead of a paper-based survey, reducing paper waste and associated carbon emissions. Participants were assured of anonymity and confidentiality, with their data solely intended for academic research. Only those who consented to participate completed the questionnaires. A total of 434 valid responses were obtained from the 550 distributed surveys, yielding a response rate of 78.91%. This exceeds the commonly recommended minimum threshold in behavioral research, which suggests that the number of subjects should typically be at least 10 times larger than the number of items being measured (Chin, 1998). A total of 116 questionnaires were excluded to ensure data quality. Of these, 57 participants were removed due to providing identical responses to all items, indicating potential response bias. Additionally, 59 participants failed the attention check question, which required selecting “disagree” for a specific item, reflecting a lack of engagement. These exclusions were necessary to maintain the integrity of the dataset and the validity of the results.

### 3.3. Statistical Instrument

Researchers have posited that partial least squares structural equation modeling (PLS-SEM) is an appropriate methodology when the objective is to prioritize predictive analysis and model complexity (Hair et al., 2021; Sarstedt et al., 2022). This study examines the mediating roles of self-expansion and psychological richness, as well as the moderating role of implicit theories of personality, making PLS-SEM an appropriate tool to estimate the complex relationships between these variables. Furthermore, the concept of tourism autobiographical memory can be regarded as a second-order structure, thereby providing an appropriate foundation for PLS-SEM (Hair et al., 2023). In addition, the unexplored mechanism by which tourism autobiographical memory influences TERB positions this work as an exploratory investigation aimed at testing the theoretical framework from a predictive perspective (Hair et al., 2023). Therefore, PLS-SEM is an appropriate method to use in this study. Data analysis was performed using SmartPLS 4 (Ringle et al., 2024).

## 4. Results

### 4.1. Descriptive Analysis

Among the 434 survey respondents, 176 (40.6%) were male and 258 (59.4%) were female. The age distribution was as follows: 30 participants (6.9%) were aged 18–19, 164 (37.8%) were aged 20–29, 121 (27.9%) were between 30 and 39 years old, 79 (18.2%) were aged 40–49, 38 (8.7%) were between 50 and 59 years old, and 2 respondents (0.5%) were 60 years old or older. In terms of educational background, 17 respondents (3.9%) held a master’s degree or higher, 245 (56.5%) had a bachelor’s degree, and 172 (39.6%) had a high school diploma or lower. The detailed demographic profiles are presented in Table 1.

### 4.2. Common Method Bias Test

The questionnaires in this study were self-reported by participants, which could have introduced a common method bias. To assess this potential common method bias, we conducted Harman’s single-factor test (Podsakoff et al., 2012). The analysis showed that the first factor explained 20.91% of the variance, which is below the 50% threshold. We also conducted a full-collinearity test and found that the variance inflation factor (VIF) values for all constructs in the inner model ranged from 1.12 to 1.84, well within the acceptable limit of 3.3 (Kock, 2015). These results indicate that our study is not significantly affected by common method biases.

### 4.3. Measurement Model

The measurement model was rigorously evaluated through a series of reliability and validity tests. Reliability was assessed using composite reliability (CR) and Cronbach’s alpha, both of which exceeded the recommended threshold of ≥0.70 (Hair et al., 2021). To ensure the robustness of the model, validity testing included both convergent and discriminant validity. Convergent validity was assessed by evaluating the average variance extracted (AVE) and factor loadings of the items. According to established criteria, AVE values should be ≥0.50 and factor loadings should exceed 0.70 (Hair et al., 2021). It is noteworthy that item 5 of the tourism autobiographical memory rehearsal scale was excluded from the model test due to a factor loading of 0.401. The remaining AVE values were all above 0.5, confirming the construct reliability of the model, as detailed in Table 2. Discriminant validity was first tested using the Heterotrait-Monotrait ratio (HTMT) of correlations, with values less than 0.85 considered acceptable (Henseler et al., 2015). As shown in Table 3, all HTMT values were within this acceptable range, confirming the discriminant validity of the constructs. Additional validation was conducted using the Fornell-Larcker criterion (Fornell & Larcker, 1981), which revealed that the square roots of all AVE values were greater than 0.8 and exceeded the internal correlation between the constructs, further confirming discriminant validity. Table 4 presents the weights and significance levels of the measurements for each first-order construct. The significance of these weights was determined through a 5000 resample bootstrap procedure, with results indicating that all indicator weights were significant at the *p* < 0.001 level (Hair et al., 2023). This underscores the relative importance of the formative indicators in constructing the second-order constructs. Moreover, multicollinearity was not a concern, as the variance inflation factor (VIF) values for all variables were below the critical threshold of 3.5 (Hair et al., 2023). The model fit was evaluated using the standardized root mean square residual (SRMR). The SRMR value obtained in this study was 0.063, which is below the generally accepted cutoff value of 0.08 (Henseler et al., 2015). Furthermore, we adhered to the guidelines set forth by Dijkstra and Henseler (2015). They put forth two metrics of model fit in PLS-SEM: d_ULS (squared Euclidean distance) and d_G (geodesic distance). These metrics serve as measures of discrepancy in PLS-SEM. The present result, consistent with Henseler et al. (2016), shows that both d_ULS and d_G are below the 95% bootstrapped quantile (HI 95% of d_ULS and HI 95% of d_G). These results confirm that the data fit the model well.

### 4.4. Structural Model Test

Table 5 presents the results of the bootstrapping procedure with 5000 samples, path coefficients, and t-values for each path. As hypothesized, tourism autobiographical memory did not have a significant positive effect on TERB (*β* = 0.048, *p* > 0.05); H1 was not supported. The relationship between tourism autobiographical memory and self-expansion was statistically significant (*β* = 0.490, *p* < 0.001), confirming H2. In support of H3, tourism autobiographical memory was positively associated with psychological richness (*β* = 0.409, *p* < 0.001).

**Figure 2:**
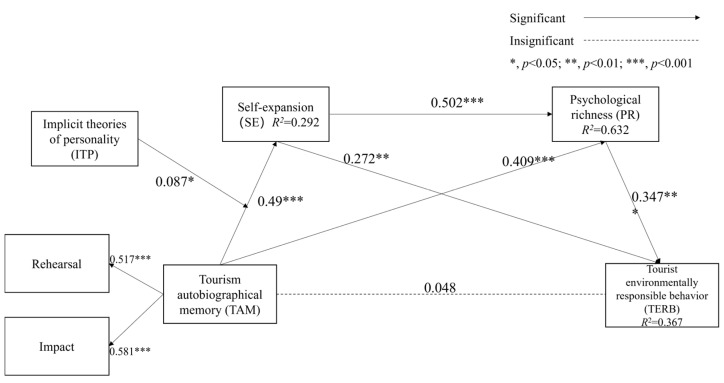
Results of the hypothetical model.

Table 5 shows the results of the mediation effects of self-expansion and psychological richness. The indirect effect of tourism autobiographical memory on TERB through self-expansion was 0.133, and the 95% confidence interval was (0.055, 0.220). Hypothesis 4 was verified. The indirect effect of tourism autobiographical memory on TERB through psychological richness was 0.142, and the 95% confidence interval was (0.74, 0.215). Hypothesis 5 was verified. The indirect effect of tourism autobiographical memory on TERB through self-expansion and psychological richness was 0.085, and the 95% confidence interval was (0.047, 0.129). Hypothesis 6 was verified. The 95% confidence interval was not zero for all paths. This indicates that all mediation effects were significant. In addition, the beta of the direct effect of tourism autobiographical memory on TERB was 0.048 (*p* > 0.5). Thus, tourism autobiographical memory did not have a significant direct effect on TERB, and the chain mediation effects were full positive mediation effects (see Figure 2). The association between tourism autobiographical memory and TERB was mediated independently and jointly by self-expansion and psychological richness. Furthermore, the moderating impact of implicit theories of personality on the relationship between tourism autobiographical memory and self-expansion was significant (*β* = 0.087, *p* < 0.05), so H7 was supported. To evaluate the predictive relevance of our model, we employed the Stone-Geisser test (Q^2^) (Geisser, 1974; Stone, 1974). The Q^2^ values yielded results of 0.02, 0.15, and 0.35, indicating small, medium, and high predictive relevance, respectively. Specifically, the Q^2^ values for the psychological richness, self-expansion, and TERB were 0.423, 0.249, and 0.284, respectively. These values are all greater than 0, indicating that each of these constructs has a medium level of predictive relevance.

To more effectively illustrate the moderating influence of implicit personality theory on the pivotal interaction between tourism autobiographical memory and self-expansion, we referenced prior studies (Greene et al., 2010; Shen et al., 2024) that classified subjects into two groups based on their mean scores (standard deviation above or below the mean) on implicit personality theory. The first group (above the mean) had a tendency toward incremental theory, while the second group (below the mean) had a tendency toward entity theory. The predictive effect of TAM on SE was then examined in these two groups (see Table 6). The results indicate that incremental theorists’ TAM predicted greater SE compared to those of entity theorists (the *β*_entity theorists_ = 0.404, *p* < 0.001, the *β*_incremental theorists_ = 0.575, *p* < 0.001).

## 5. Discussion and Implications

### 5.1. General Discussion

Drawing from self-expansion theory (Schultz, 2001), the present study presented a moderated multiple mediation model with self-expansion and psychological richness as the mediating variables and implicit theories of personality as the moderating variable. The research results support the majority of the research hypotheses and have some theoretical and practical implications for further exploring the association between tourism autobiographical memory and TERB.

The insignificant direct effect of tourism autobiographical memory on TERB in the present study may be due to the fact that the tourism autobiographical memories examined were not destination-specific, unlike the memories examined in previous studies. For example, previous studies have focused on specific memories associated with tourist destinations such as Antarctica (Cajiao et al., 2023), as well as memories related to wildlife (Buckley, 2022), with a particular emphasis on the environmental friendliness of tourist destinations. In contrast, the findings of this study highlight the mediating pathway of general tourist autobiographical memories on TERB, offering a novel perspective on the effect of tourist autobiographical memories on TERB.

The study demonstrated that the positive effect of tourism autobiographical memory on TERB is mediated by self-expansion. Our findings suggest that when individuals reflect on their past travels, these memories contribute to an expansion of their self. This self-expansion, as outlined in self-expansion theory, involves the integration of new perspectives and experiences into one’s self, thereby expanding the boundaries of the self to include not only a deeper connection with other people, but also with the natural environment (Nolan & Schultz, 2015; Schultz, 2001). As tourists internalize these expanded boundaries, their sense of self becomes more intertwined with the environments and cultures they encounter. This expanded sense of self fosters a sense of responsibility for the preservation of these elements, motivating tourists to engage in TERB (Nolan & Schultz, 2015). The expanded self, now encompassing diverse human relationships and natural connections, drives actions that reflect a commitment to protecting the world that has become part of their expanded self (Schultz, 2001; Tang et al., 2017). This finding aligns with previous research showing that strong travel memories can not only increase awareness but also inspire the adoption of long-lasting environmentally friendly attitudes and behaviors (Buckley, 2022; Cajiao et al., 2023). Moreover, in addition to self-expansion, powerful travel memories can also evoke emotional experiences such as awe and “eco-guilt” (Bahja & Hancer, 2021; Stellar, 2021), which can subsequently lead to TERB.

The study showed that the positive effect of tourism autobiographical memory on TERB is mediated by psychological richness. When tourists recall travel experiences that were particularly enriching, whether through exposure to new cultures, challenging situations, or profound encounters with nature, these memories contribute to a psychologically rich life (Oishi et al., 2021). This enriched perspective makes individuals more attuned to the value of preserving the environments and communities with which they have interacted during their travels (Oishi & Westgate, 2022). The influence of psychological richness as a mediator indicates that it is not merely the content of memories, but rather the depth and complexity of the experiences they represent, that drives TERB (Buttrick et al., 2023; Oishi et al., 2024). Tourists whose memories evoke a sense of psychological richness are more prone to view the world through a lens that values diversity, complexity, and interconnectedness, which in turn motivates actions consistent with environmental sustainability (Buckley, 2022; Oishi et al., 2024). In addition, unforgettable travel memories are not always pleasant (Kim & Chen, 2019), and research suggests that negative emotions also promote environmental action, because these negative feelings may increase tourists’ concern about environmental problems such as global warming (Massingham et al., 2019). Negative emotional travel memories may also promote psychological richness, which focuses on varied and complex experiences rather than pleasant and meaningful ones (Oishi & Westgate, 2022).

The results of our study indicate that the positive effect of tourist autobiographical memory on TERB is sequentially mediated by self-expansion and psychological richness. These findings are grounded in the principles of self-expansion theory (Nolan & Schultz, 2015). In accordance with this theory, individuals are driven to expand their self by integrating novel and meaningful experiences (Aron et al., 2004). When tourists recall impactful travel experiences, these memories contribute to self-expansion by broadening their sense of self to include perspectives from others and from nature (Schultz, 2001). As this expanded self develops, it fosters psychological richness (Oishi et al., 2024). Research has demonstrated that individuals who exhibit high levels of psychological richness are more likely to accept an understanding of the complexity of their environment and engage in action (Wei et al., 2023), which enhances the likelihood of engaging in TERB.

The results of this study suggest that implicit theories of personality exert the moderating influence on the association between tourist autobiographical memory and self-expansion. Specifically, individuals who hold an incremental theory (who believe in the malleability of personality traits) are more likely to integrate diverse travel experiences into their self (Yeager et al., 2014), thereby enhancing self-expansion. In contrast, those with an entity theory (who believe that personality traits are fixed) may be less inclined to utilize these memories for personal growth (Steimer & Mata, 2016). This discovery is in line with prior research indicating that people with an incremental theory are more open to new experiences and more likely to perceive new things and challenges as opportunities for self-development (Mattingly et al., 2019). This suggests that the impact of travel memories on self-expansion is stronger for those who hold an incremental theory.

### 5.2. Theoretical Implications

This study contributes to the existing literature on the application of self-expansion theory in the context of tourism (Nolan & Schultz, 2015). The study illustrates how tourist autobiographical memories can facilitate self-expansion, a process that has traditionally been examined in the context of interpersonal relationships (Aron et al., 2004). This is achieved by broadening the human and natural perspectives within the self-boundary (Schultz, 2001). This introduces a new dimension to the theory, emphasizing its applicability beyond personal relationships to include interactions with environments and others during and after travel. By identifying self-expansion and psychological richness as sequential mediators between tourist autobiographical memory and TERB, this study introduces a nuanced understanding of how personal growth processes influence sustainable behaviors. This model enriches existing tourism behavior theories by linking psychological mechanisms with environmentally responsible outcomes, offering a more comprehensive explanation of how and why tourist autobiographical memories shape TERB. The study highlights the moderating influence of implicit personality theories on the association between tourist autobiographical memory and self-expansion. This contribution underscores the importance of individual differences in cognitive frameworks. The findings bridge tourism studies with psychological theories on mindset, suggesting that tourists’ implicit beliefs are critical in shaping the impact of their travel memories. This research contributes to the emerging concept of psychological richness by linking it to self-expansion and TERB. Our study corroborates and extends the findings of Oishi et al. (2021) regarding the positive impact of travel experiences on psychological richness. Furthermore, it proposes that psychological richness plays a crucial role in motivating environmentally responsible behaviors, thereby broadening the theoretical understanding of what drives environmentally responsible tourism. In conclusion, the current study presents theoretical contributions by establishing a link between self-expansion, psychological richness, and implicit personality theories and TERB. This provides a comprehensive, interdisciplinary framework for understanding how tourist autobiographical memories influence TERB.

### 5.3. Practical Implications

In light of the role of tourism autobiographical memory in facilitating self-expansion, it may be beneficial for tourism marketers to consider integrating reflective practices into their travel activities. For instance, tour guides could utilize persuasive communication methods, such as storytelling and drawing, to strengthen the emotional link with tourists and facilitate the internalization of their travel experiences into autobiographical memory (Soulard et al., 2021). By prompting tourists to engage in active reflection on their trip, which increases the probability that the travel memory will lead to voluntary behavioral change, these practices can enhance the impact of the travel memory on tourists’ long-term behavior, including environmentally responsible behavior (Cajiao et al., 2023; Jorgenson et al., 2019). This study highlights the importance of self-expansion and psychological richness in driving environmentally responsible behavior. Tour operators and destinations may utilize this finding to gain new insights into their programs. In addition to the conventional approach of designing enjoyable tourism experiences, it is now feasible to develop tourism experiences that enrich and shift the perspectives of tourists (Oishi et al., 2021; Oishi et al., 2024). By offering opportunities for deep cultural immersion, meaningful interactions with local communities, and engagement with natural environments, the tourism industry can help facilitate self-expansion and psychological richness in tourists, which can lead to more TERB. For policy makers, sustainable development can be promoted by focusing on the quality and depth of the tourist experience, rather than simply increasing the number of tourists which can lead to more sustainable tourism development that benefits both the natural environment and local livelihoods. The findings align with the Sustainable Development Goals (SDG) (Katila et al., 2019) by demonstrating how TERB, such as minimizing waste, conserving resources, and reducing carbon footprints, can motivate sustainable consumption and climate-conscious actions (Qiu et al., 2023). This is evidenced by the fact that TAM and self-expansion can serve as powerful motivators for such behaviors. Furthermore, the study lends support to SDG 12 (Responsible Consumption and Production) and 15 (Life on Land) by underscoring the function of TERB in cultivating dedication to the preservation of biodiversity and the safeguarding of natural ecosystems (Qiu et al., 2023; Tang et al., 2017). These interconnections underscore the potential for psychological motivators to be harnessed in order to align tourism practices with global sustainability objectives.

### 5.4. Limitations and Future Research Directions

While the present study offers meaningful insights into the relationships between tourist autobiographical memory, self-expansion, psychological richness, and TERB, it also has several limitations. First, as the study employs self-reporting, it is susceptible to biases inherent in the reporting process, including recall bias. Tourists may overestimate their TERB or the impact of their travel memories. Future research could include more objective measures of behavior, such as tracking actual sustainable practices during and after travel. Second, this study may not fully account for the influence of contextual factors, such as the type of destination and the nature of the travel experience (e.g., adventure or cultural tourism). These factors could significantly influence the extent to which travel memories contribute to self-expansion and TERB. Future research could examine how different travel contexts and settings influence these dynamics. Third, the use of hotel-based tourists as the primary sample may introduce selection bias by excluding budget travelers and independent tourists who may exhibit different environmentally responsible behaviors, which may affect the generalizability of the results. Thus, future study could consider broader sampling methods to capture a more diverse range of tourists. Fourth, the potential role of age was not investigated as a moderating variable in this study. Age-related differences may influence how tourists process autobiographical memories and engage in TERB due to differences in life stage, travel motivations, and psychological needs. To gain a more detailed understanding of these relationships, future research should examine the moderating role of age. Fifth, it is important to note that this study was conducted exclusively in China, which may have implications for the generalizability of the results to other cultural contexts. To enhance the applicability of the findings across diverse cultural settings, future research should include cross-cultural comparisons to assess the universality or variability of the observed relationships. Finally, incorporating qualitative methods alongside quantitative measures could enrich the understanding of how tourists interpret and reflect on their travel experiences. In-depth interviews could provide nuanced insights into the personal meaning of travel memories and their impact on behavior.

## 6. Conclusions

This study integrates self-expansion theory, psychological richness, and autobiographical memory to examine the psychological processes underlying TERB. The findings, based on data from 434 participants in China, indicate that tourism autobiographical memory serves as a catalyst for self-expansion, which subsequently enhances psychological richness and motivates TERB. Moreover, the moderating effect of implicit personality theories highlights the impact of individual differences, with incremental beliefs reinforcing the association between travel memories and self-expansion. These results expand the applicability of self-expansion theory and provide a novel perspective on the intrinsic factors influencing sustainable tourism behavior. This research enhances theoretical frameworks and offers actionable insights, such as the creation of travel experiences that facilitate personal growth and environmental engagement. The findings support the development of culturally informed strategies to foster sustainability in tourism, aligning practices with global environmental goals.

## Figures and Tables

**Table 1:** Demographic profiles of respondents (N = 434).

Variable	Category	Frequency	Percent
Gender	Male	176	40.6%
	Female	258	59.4%
Age (y)	18–19	30	6.9%
	20–29	164	37.8%
	30–39	121	27.9%
	49–49	79	18.2%
	50–59	38	8.7%
	Above 60	2	0.5%
Education	High school or below	172	39.6%
	Undergraduate	245	56.5%
	Master or above	17	3.9%

**Table 2:** Reliability and convergent validity results.

Items	Mean	SD	Factor Loadings	Cronbach’s Alpha	Composite Reliability	AVE
TAMR				0.862	0.906	0.670
TAMR1	3.569	1.782	0.868			
TAMR2	3.783	1.794	0.865			
TAMR3	2.972	1.723	0.739			
TAMR4	3.378	1.699	0.822			
TAMR5 *	1.795	1.054	0.411			
TAMI				0.898	0.936	0.831
TAMI1	4.468	1.624	0.803			
TAMI2	4.235	1.595	0.832			
TAMI3	4.053	1.641	0.813			
PR				0.934	0.945	0.679
PR1	4.839	1.383	0.794			
PR2	4.687	1.381	0.860			
PR3	4.594	1.434	0.824			
PR4	4.608	1.404	0.870			
PR5	4.452	1.420	0.860			
PR6	4.412	1.417	0.835			
PR7	4.553	1.386	0.828			
PR8	4.553	1.407	0.820			
PR9	4.438	1.395	0.838			
PR10	4.445	1.516	0.794			
PR11	4.574	1.459	0.832			
PR12	4.355	1.460	0.772			
SE				0.951	0.962	0.866
SE1	4.505	1.297	0.901			
SE2	4.507	1.244	0.930			
SE3	4.548	1.305	0.935			
SE4	4.530	1.262	0.940			
SE5	4.601	1.281	0.947			
TERB				0.910	0.914	0.788
TERB1	4.871	1.281	0.910			
TERB2	4.896	1.293	0.908			
TERB3	4.696	1.293	0.868			
TERB4	4.894	1.301	0.864			
ITP				0.929	0.932	0.876
ITP1	3.751	1.462	0.927			
ITP2	3.696	1.422	0.950			
ITP3	3.712	1.441	0.930			

Note: TAMR = tourism autobiographical memory rehearsal; TAMI = tourism autobiographical memory impact; PR = psychological richness; SE = self-expansion; TERB = tourist environmentally responsible behaviors; ITP = implicit theories of personality; * Presented on a 4-point scale and deleted in measurement model test.

**Table 3:** Discriminant validity and intercorrelations.

	1	2	3	4	5	6
Heterotrait-Monotrait Ratio						
1 ITP						
2 TERB	0.33					
3 SE	0.188	0.581				
4 TAMI	0.183	0.463	0.512			
5 TAMR	0.079	0.366	0.511	0.751		
6 PR	0.223	0.612	0.743	0.698	0.62	
Fornell-Larcker criterion						
1 ITP	**0.936**					
2 TERB	0.303	**0.888**				
3 SE	0.178	0.545	**0.931**			
4 TAMI	0.167	0.42	0.476	**0.911**		
5 TAMR	0.071	0.337	0.47	0.666	**0.819**	
6 PR	0.211	0.573	0.714	0.649	0.569	**0.824**

Note: Bolded values are square roots of AVEs, while values below the bold values indicate the internal correlation between the constructs; TAMR = tourism autobiographical memory rehearsal; TAMI = tourism autobiographical memory impact; PR = psychological richness; SE = self-expansion; TERB = tourist environmentally responsible behaviors; ITP= implicit theories of personality.

**Table 4:** Assessment of second-order construct.

Construct	Indicators	Weight	t-Value (*p*-Value)	VIF
Tourism autobiographical memory	Rehearsal	0.517	34.621 (0.000)	1.758
	Impact	0.581	35.046 (0.000)	1.748

**Table 5:** Structural model results.

Relationship	Beta	SD	*t*-Value	CI Lower	CI Upper	Remarks
H1: TAM→TERB	0.048	0.053	0.909	−0.041	0.136	Not Supported
H2: TAM→SE	0.490	0.045	10.965 ***	0.415	0.562	Supported
H3: TAM→PR	0.409	0.046	8.965 ***	0.333	0.481	Supported
SE→TERB	0.272	0.093	2.916 **	0.120	0.248	-
PR→TERB	0.347	0.092	3.757 ***	0.192	0.496	-
SE→PR	0.502	0.049	10.21 ***	0.423	0.584	-
H4: TAM→SE→TERB	0.133	0.050	2.678 **	0.055	0.220	Supported
H5: TAM→PR→TERB	0.142	0.043	3.277 ***	0.074	0.215	Supported
H6: TAM→SE→PR→TERB	0.085	0.025	3.418 ***	0.047	0.129	Supported
H7: TAM × ITP→SE	0.087	0.044	1.973 *	0.017	0.161	Supported

Note: *, *p* < 0.05; **, *p* < 0.01; ***, *p* < 0.001; TAM = tourism autobiographical memory; PR = psychological richness; SE = self-expansion; TERB = tourist environmentally responsible behaviors; ITP= implicit theories of personality.

**Table 6:** The effect value of different ITP.

Implicit Theories of Personality	Beta	*t*-Value	CI Lower	CI Upper
M + SD	0.575	9.736 ***	0.472	0.668
M − SD	0.404	5.983 ***	0.291	0.513

Note: ***, *p* < 0.001.

## Data Availability

Data will be made available on request.

## Appendix A

**Table A1:** The items of the constructs.

Constructs	Items
Tourism autobiographical memory rehearsal	1 Since it happened, I have talked about this event.
	2 Since it happened, I have thought about this event.
	3 Since it happened, I have written about this event to others (e.g., email, WeChat, letter, text).
	4 As I remember the event, I can feel now the emotions I felt then. 5 As I recall them now, I would rate the emotions I experienced during the event as * (0 = neutral, 1 = mildly positive or negative, 2 = positive or negative, and 3 = extremely positive or negative).
Tourism autobiographical memory impact	1 As I remember the event, it comes to me in words or in pictures as a coherent story or episode and not as an isolated fact, observation, or scene.
	2 This memory is significant in my life because it imparts an important message for me or represents an anchor, critical juncture, or turning point.
	3 This memory has consequences for my life because it influenced my behavior, thoughts, or feelings in noticeable ways.
Self-expansion	1 Do you feel a greater awareness of things?
	2 Do you feel an increase in your ability to accomplish new things?
	3 How much do you feel that you have a larger perspective on things?
	4 How much has doing the previous activity resulted in your learning new things?
	5 How much has doing the previous activity increased your knowledge?
Psychological richness	1 My life has been psychologically rich.
	2 My life has been experientially rich.
	3 My life has been emotionally rich.
	4 I have had a lot of interesting experiences.
	5 I have had a lot of novel experiences.
	6 My life has been full of unique, unusual experiences.
	7 My life consists of rich, intense moments.
	8 I experience a full range of emotions via first-hand experiences such as travel and attending concerts.
	9 I have a lot of personal stories to tell others.
	10 On my deathbed, I am likely to say “I had an interesting life”.
	11 On my deathbed, I am likely to say “I have seen and learned a lot”.
	12 My life would make a good novel or movie.
Tourist environmentally responsible behavior	1 I discuss environmental protection issues of the destination with companions.
	2 I try to convince companions to adopt positive behaviors in the environment of this destination.
	3 I report activities damaging the environment of the destination.
	4 When I see trash in the destination, I pick it up.
Implicit theories of personality ^#^	1 The kind of person someone is something very basic about them and it can’t be changed very much.
	2 People can do things differently, but the important parts of who they are can’t really be changed.
	3 Everyone is a certain kind of person and there is not much that can be done to really change that.

Note: * Presented on a 4-point scale and deleted in measurement model test; ^#^ Represents reverse scoring.
